# The prevalence and risk of developing major depression among individuals with subthreshold depression in the general population

**DOI:** 10.1017/S0033291722000241

**Published:** 2023-06

**Authors:** Ruibin Zhang, Xiaoling Peng, Xiaoqi Song, Jixin Long, Chanyu Wang, Chichen Zhang, Ruiwang Huang, Tatia M. C. Lee

**Affiliations:** 1 Laboratory of Cognitive Control and Brain Healthy, Department of Psychology, School of Public Health, Southern Medical University, Guangzhou, China; 2 Department of Psychiatry, Zhujiang Hospital, Southern Medical University, Guangzhou, China; 3 Guangzhou Cana School, Guangzhou 510515, China; 4 School of Management, Southern Medical University, Guangzhou, China; 5 School of Psychology, South China Normal University, Guangzhou, China; 6 State Key Laboratory of Brain and Cognitive Sciences, The University of Hong Kong, Hong Kong, SAR China; 7 Laboratory of Neuropsychology and Human Neuroscience, The University of Hong Kong, Hong Kong, SAR China; 8 Center for Brain Science and Brain-Inspired Intelligence, Guangdong-Hong Kong-Macao Greater Bay Area, Guangzhou, China

**Keywords:** Depression, meta-analysis, mood, prevalence, spectrum disorder

## Abstract

**Background:**

Subthreshold depression could be a significant precursor to and a risk factor for major depression. However, reliable estimates of the prevalence and its contribution to developing major depression under different terminologies depicting subthreshold depression have to be established.

**Methods:**

By searching PubMed and Web of Science using predefined inclusion criteria, we included 1 129 969 individuals from 113 studies conducted. The prevalence estimates were calculated using the random effect model. The incidence risk ratio (IRR) was estimated by measuring the ratio of individuals with subthreshold depression who developed major depression compared to that of non-depressed individuals from 19 studies (88, 882 individuals).

**Results:**

No significant difference in the prevalence among the different terminologies depicting subthreshold depression (*Q* = 1.96, *p* = 0.5801) was found. By pooling the prevalence estimates of subthreshold depression in 113 studies, we obtained a summary prevalence of 11.02% [95% confidence interval (CI) 9.78–12.33%]. The youth group had the highest prevalence (14.17%, 95% CI 8.82–20.55%), followed by the elderly group (12.95%, 95% CI 11.41-14.58%) and the adult group (8.92%, 95% CI 7.51–10.45%). Further analysis of 19 studies' incidence rates showed individuals with subthreshold depression had an increased risk of developing major depression (IRR = 2.95, 95% CI 2.33–3.73), and the term minor depression showed the highest IRR compared with other terms (IRR = 3.97, 95% CI 3.17–4.96).

**Conclusions:**

Depression could be a spectrum disorder, with subthreshold depression being a significant precursor to and a risk factor for major depression. Proactive management of subthreshold depression could be effective for managing the increasing prevalence of major depression.

## Introduction

Major depression is one of the leading causes of mortality worldwide (Vos et al., 2017). Under the current diagnostic systems, the classification of depressive disorders is categorical (Uher, Payne, Pavlova, & Perlis, 2014). However, researchers have proposed a dimensional approach in which depressive disorders are considered to exist along a spectrum of increasing severity (McElroy, Guerdjikova, & Romo-Nava, 2021). From the spectrum perspective, individuals who experience clinically relevant depressive symptoms that do not meet the diagnosis criteria for major depression could be diagnosed with minor or subthreshold depression (Kroenke, 2017; Rodríguez, Nuevo, Chatterji, & Ayuso-Mateos, 2012).

Although the symptoms of subthreshold depression are less severe than the symptoms of major depression, subthreshold depression is associated with a greater health service burden than major depression (Liu et al., 2020) due to its higher prevalence rate in the population compared with major depression (Kroenke, 2017; Topuzoğlu et al., 2015). However, the estimated prevalence of subthreshold depression varies across studies from 1.50% (Rivas, Nuevo, & Ayuso-Mateos, 2011) to 41.27% (Pickett et al., 2014). Moreover, existing studies report conflicting findings regarding how the estimated prevalence of subthreshold depression varies by sex, age, or other characteristics (Crockett, Martínez, & Jiménez-Molina, 2020; Curran, Rosato, Ferry, & Leavey, 2020). Reliable estimates of subthreshold depression prevalence are important to informing efforts to identify, treat, and prevent the causes of emotional distress.

Recent studies have shown that individuals with subthreshold depression experience persistent depressive symptoms at 12-month follow-up, and a third to half of these individuals report moderate functional impairment (Kroenke, 2006; Lee et al., 2019). Furthermore, studies have consistently demonstrated that individuals with subthreshold depression have an increased risk of developing major depression (Lee et al., 2019; Tuithof et al., 2018). In a narrative review of 20 published studies, Cuijpers and Smit (2004) found that individuals with subthreshold depression have a risk ratio of 1.15 to 9.73 for developing major depression compared with individuals without depression in the general population. Notably, few studies have calculated a pooled estimate that quantifies the magnitude of this risk across multiple studies. After calculating the incidence rate ratio (IRR) of major depression among people with subthreshold depression relative to non-depressed individuals across 16 studies, Lee et al. (2019) suggested that, compared with individuals without depressive symptoms, those with subthreshold depression are 1.95 times more likely to develop major depression. However, the most recent study included in the paper is from 2016. Since then, there have been several studies with large sample sizes (e.g. Oh *et al*. 2021; Sigström, Waern, Gudmundsson, Skoog, & Östling, 2018; Tuithof *et al*. 2018). Therefore, there is a need for updated research covering the latest studies to revise the ratio of individuals with subthreshold depression who develop major depression.

Notably, there are different terms depicting subthreshold depression, which might originate from the various definitions of the subthreshold condition. Generally, minor depression was defined according to diagnosis criteria (i.e. DSM-IV), while for subthreshold depression, also called subclinical depression or subsyndromal symptomatic depression, between 2 and 5 depressive symptoms were required for the diagnosis, and a minimum duration of 2 weeks. This heterogeneity of definition leads to a lack of comparability of studies with regard to the identification and management of subthreshold depressive disorders. For example, Baumeister and Morar (2008) found higher prevalence rates based on the symptom counts alone, compared with additional categorically and dimensionally operationalized clinical significance criteria. Their results demonstrate that the operationalization of subthreshold depression might impact the estimation of its prevalence. Moreover, agreement in the definition and conceptualization of subthreshold depression is also needed in order to achieve a better understanding of the boundaries of depression (Rodríguez et al., 2012). Thus, it is pertinent to investigate whether the different terms characterizing subthreshold depression show different prevalence and different incidence risk ratio (IRR) of subthreshold depression developing into major depression.

The aim of this study was two folds: (1) to chart the estimated prevalence of subthreshold depression and whether it is significantly different among the various terms depicting subthreshold depression in the general population and (2) to qualitatively summarize the risk of subthreshold depression developing into major depression as identified in the latest studies. To achieve these two goals, we conducted a systematic review and meta-analysis of published studies of subthreshold depression among the general population. Additional stratified subgroup analyses were performed to investigate the effects of sociodemographic (i.e. age) and methodological factors (i.e. sample type) on mean estimates. Beyond that, we also estimated the prevalence of major depression if there were data available. With these analyses, we sought to provide evidence for whether depression is better expressed as a spectrum on which subthreshold depression coexists with major depression.

## Methods

### Search strategy and study eligibility

Our search strategy followed the guidelines described in the PRISMA statement (Moher, Liberati, Tetzlaff, & Altman, 2009; Page et al., 2021). On 30 January 2021, we conducted a systematic search on PubMed and Web of Science using keywords in the following search string: ‘minor depression’ OR ‘subclinical depression’ OR ‘subsyndromal depression’ OR ‘subthreshold depression’ OR ‘subthreshold depressive symptoms’ OR ‘subclinical depressive conditions.’ In addition, we used the snowball search method to identify any additional studies reporting the prevalence of subthreshold depression.

**Fig. 1.**
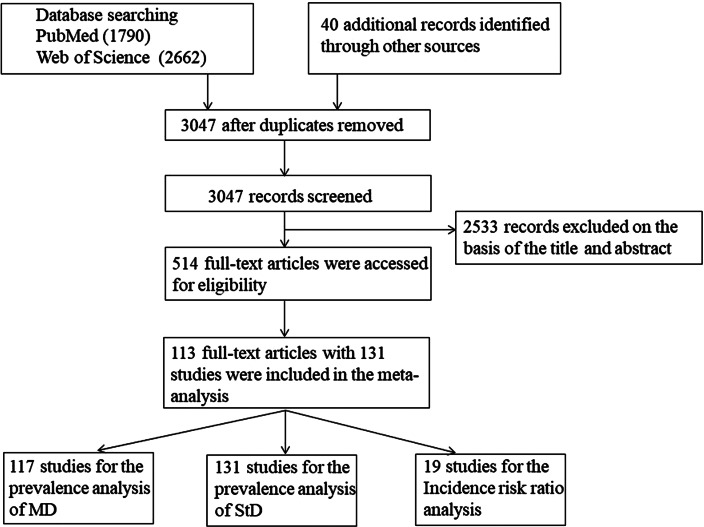
Flowchart of study selection and identification following the PRISMA statement. PRISMA, Preferred Reporting Items for Systematic reviews and Meta-Analyses. MD, major depression; StD, subthreshold depression.

The inclusion criteria were as follows: Papers had to be original observational and epidemiological studies examining the definition, prevalence, and associated characteristics of minor/subthreshold depression in the general population. They also had to come from the community and primary care settings, have been published in peer-reviewed journals, involve subjects without organic disorders or cognitive impairments, and be written in English. We also controlled for statistical non-independence by excluding overlapping studies reporting on the same cohort, and we excluded overlapping studies with less rigorous case definitions of subthreshold depression (e.g. subthreshold depression defined using the depression rating scale). When overlapping studies employed similar case definitions, we retained the study with greater coverage of the overall cohort sample. Figure 1 shows the detailed flowchart. In the end, 113 studies with 131 samples were entered into data analysis (see online Supplementary Table S1 in the supplemental data). Among the 113 studies, 19 (see online Supplementary Table S2 in the supplemental data) used available data in a longitudinal design to estimate the risk of subthreshold depression developing into major depression.

### Data extraction and quality assessment

We extracted the following information from the included studies using EpiData (Classic) Entry v3.1 (https://epidata.dk/): study design; geographic location (country); years of survey; sample size; average age of participants (including age range), if available; number of female participants; diagnostic or screening method used; and outcome definition (i.e. specific diagnostic criteria or screening instrument cutoff) (see online Supplemental data). We also reported prevalence estimates for subthreshold depression and major depression, as well as the gender-specific prevalence of subthreshold depression. In the case of longitudinal studies, where data were available, the average length of follow-up in years, the percentage lost to follow-up, and the number of the subjects without subthreshold depression who developed major depression were also extracted. We coded each included study into three age groups based on the mean age or age range following previous studies (Cuijpers et al., 2020; Wang, Tong, Li, Li, & Li, 2021): youth (< 18 years), adult (18–60 years), and elderly (> 60 years).

In addition to the above-mentioned information, we coded the risk of bias in these nonrandomized studies using a modified version of the Newcastle-Ottawa scale (Wells et al., 2000). This scale comprises five items: sample representativeness, sample size, non-respondents, ascertainment of subthreshold depression, and quality of descriptive statistics reporting (see online Supplementary Table S3 in the supplemental data). If the total score on the Newcastle-Ottawa scale was greater than 3 points, the studies were judged to be at low risk of bias, whereas if the score was less than or equal to 3 points, the studies were coded as being at high risk of bias.

### Data synthesis and analysis

#### Prevalence estimates of subthreshold depression and major depression

Prevalence estimates for subthreshold depression and major depression were calculated by pooling the study-specific estimates using random-effects meta-analyses that accounted for between-study heterogeneity (Barendregt, Doi, Lee, Norman, & Vos, 2013). The overall period prevalence was used when studies reported point prevalence estimates made at different times in a year. The random effect model assumes that each study estimated different values from the distribution of population parameters, which would be flexible to heterogeneous effect sizes and the conservative nature of estimation (Munn, Stern, Aromataris, Lockwood, & Jordan, 2018). We assessed heterogeneity across effect sizes with Cochran's Q test, calculated as the weighted sum of squared differences between the effects of individual studies and the pooled prevalence across studies. In addition, we estimated the *I*^2^ index to quantify heterogeneity between the included studies, with values of 25%, 50%, and 75% reflecting small, medium, and large degrees of heterogeneity, respectively (Higgins & Thompson, 2002).

We performed a stratified meta-analysis with the following characteristics: search terms tapping subthreshold depression, outcome definition (see the supplemental data), continent or region, country, Newcastle-Ottawa Scale components, age, sex, and odds ratios (OR)—evaluating whether the odds of a certain outcome (e.g. depression) are the same for two groups (e.g. males and females). OR values were estimated to address whether there are significant differences between males and females. To further determine the influence of each included sample on the overall prevalence estimations, (1) we detected and removed extreme estimates (outliers) using the InfluenceAnalysis function (https://raw.githubusercontent.com/MathiasHarrer/dmetar/master/R/influence.analysis.R), and (2) we performed sensitivity analyses by serially excluding each study. To check the publication bias, we performed Egger's regression intercept test and funnel plots, which displayed confidence interval (CI) boundaries for visualizing whether the studies were distributed symmetrically within the funnel, assessing sensitivity using the leave-one-out cross-validation procedure to indicate the impact of each study on the net results.

#### Incidence risk ratio of subthreshold depression developing into major depression

If the data were available (longitudinal study with detailed data describing the individuals with subthreshold depression developing into major depression), we calculated IRRs (Sedgwick, 2010) for the study by dividing the incidence rate of major depression in the exposed cohort (i.e. individuals with subthreshold depression) by the incidence rate of major depression in the control cohort (i.e. non-depressed people). We used 19 studies in this step (see online Supplementary Table S2 in the supplemental data). We established the incidence rate for each cohort by dividing the total number of people diagnosed with major depression at follow-up by the total person-years of the cohort. An IRR value greater than 1 represents a likelihood of individuals with subthreshold depression having a higher risk of developing major depression compared to non-depressed individuals. In addition to pooling each study as an IRR, by extending our previous work (Zhang et al., 2021), for each study, we calculated the percentage of individuals with subthreshold depression (non-depressed) who transitioned to having major depression and performed two-sample *t* tests to identify significant differences between the two groups at *a* = 0.05.

When the data were available, we also checked the prevalence rate of major depression in the included studies. All of the data analyses were performed using R with the ‘meta’ and ‘metafor’ tools using the guidelines provided by (Harrer, Cuijpers, Furukawa, & Ebert, 2021). The forest plot was generated by the ‘forestplot’ toolbox.

## Results

### Study characteristics

In total, 1 129 969 individuals in 39 countries from 113 studies of 131 data sets were included in the analysis (see Fig. 1 and online Supplementary Table S1 in the supplemental data). The median number of participants per study was 1709 (range, 71– 237 023). Among the included studies, 25 were longitudinal, and of these, 19 were fit for estimating the IRR (see online Supplementary Table S2 in the supplemental data). The continent or region, country, diagnostic criteria, sample type (community-based or primary care), and the total Newcastle-Ottawa scores for the included studies are listed in online Supplementary Table S1 in the supplemental data. Additionally, the search terms used in characterizing subthreshold depression are also listed in online Supplementary Table S1, i.e., minor depression (67 samples), subclinical depression (four samples), subsyndromal depression (22 samples), subthreshold depression (38 samples), subthreshold depressive symptoms (0 sample), subclinical depressive conditions (0 sample) The Newcastle-Ottawa score components for all 113 studies are listed in online Supplementary Table S3 in the supplemental data.

### Prevalence of subthreshold depression

**Fig. 2.**
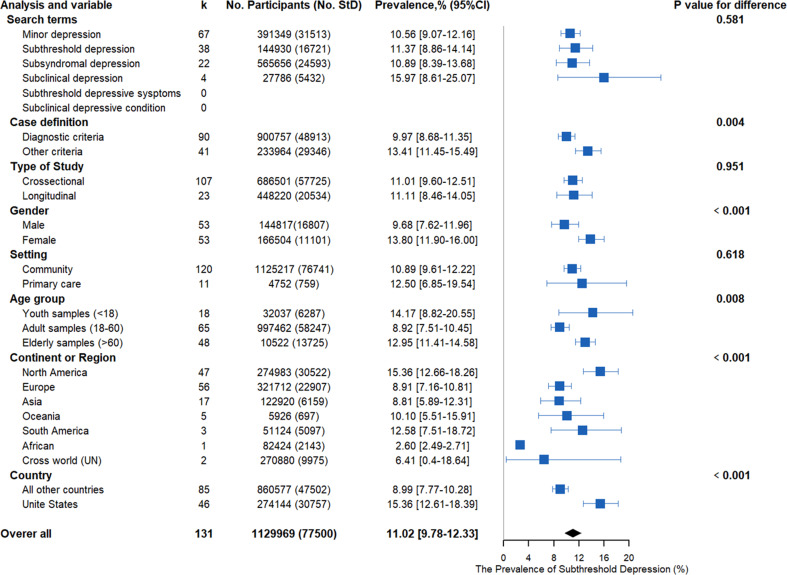
Meta-analysis of the prevalence of subthreshold depression under different subgroup analyses. StD, subthreshold depression; MD, major depression. CI, confidence interval.

**Fig. 3.**
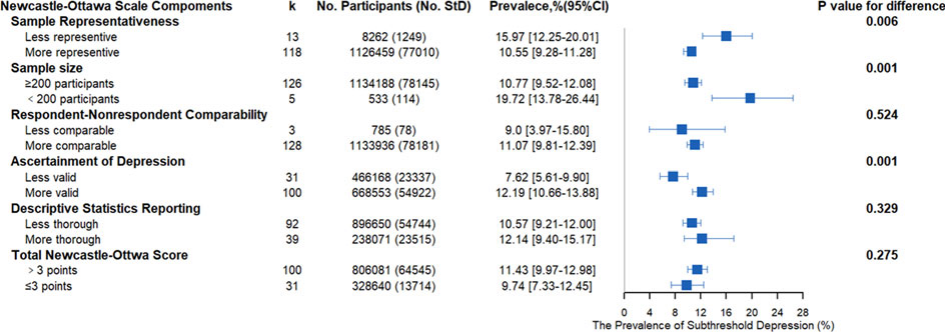
Meta-analysis of the prevalence of subthreshold depression under subgroup analysis using different diagnostic criteria.

**Fig. 4.**
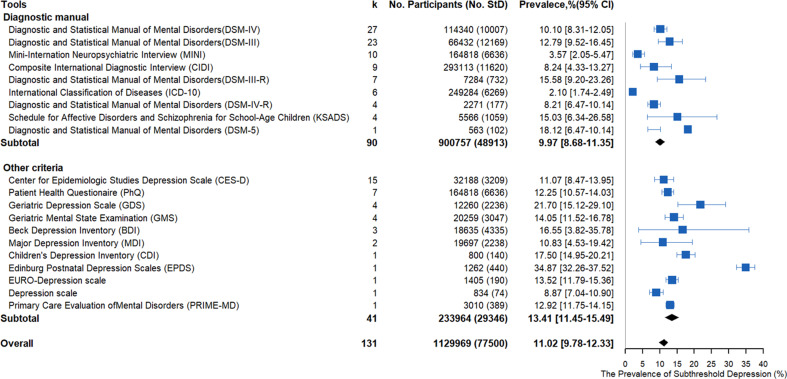
Meta-analysis of the prevalence of subthreshold depression under subgroup analysis using the Newcastle-Ottawa scale.

We first tested whether the prevalence derived from different search terms might significantly differ and found that there was no significant difference among the classes (*Q* = 1.96, *p* = 0.5801, Fig. 2 and online Supplementary Table S5 in the Supplementary Materials). Moreover, by using the leave-one-out cross-validation procedure where we excluded studies from one class at a time, we found that the estimations of prevalence were quite consistent (ranging from 10.88% to 11.48%). Therefore, we believe that it is acceptable to combine the different classes to characterize the prevalence of subthreshold depression. Then, we combined all samples from different search terms and found that the prevalence of subthreshold depression in the 113 studies ranged from 1.51% to 41.27%. Pooling the prevalence estimates of subthreshold depression of all included studies yielded a summary prevalence of 11.02% (77 500/1 129 969 individuals; 95% CI9.78–12.33%; Figs 2–4), with significant evidence of between-study heterogeneity (*Q* = 58 128.75, *τ*au^2^ = 0.0138, *I*^2^ = 99.8%, *p* < 0.001). Regarding the gender difference, females [11 101/166 504, (13.8%, 95% CI 11.90%–16.00%)] have a higher risk of experiencing subthreshold depression compared with males [16 807/144 817, (9.68%, 95% CI 7.62%–11.96%); odds ratio (OR) = 1.43, 95% CI 1.25–1.63, *t* = 5.25, *p* < 0.001; see Fig. 2 and online Supplementary Fig. S1 in the supplemental data].

We performed two steps to confirm whether the pooled effect estimate was heavily dependent on a single study. First, we detected and removed extreme prevalence (outliers) (Pickett et al., 2014), and the equivalent figures were as follows: summary prevalence 10.95% (95% CI 9.62–12.14%). Second, we performed a sensitivity analysis by leaving one experiment out each time and re-estimating the summary prevalence of subthreshold depression. No individual study affected the overall prevalence estimate; the difference between maximum and minimum was 0.28% (see online Supplementary Table S4 in the supplemental data).

Subgroup analysis of the definitions of subthreshold depression using a diagnostic manual (see the online Supplemental data) or diagnostics with other measures (e.g. self-reported questionnaires) were performed (Fig. 4) to further characterize the range of subthreshold depression prevalence estimates identified by the potential methodological factors. There was a significant difference between the two definitions (diagnostic manual *v.* other measures, *Q* = 8.01, *p* = 0.004). The summary prevalence of the studies (91 data sets) using the classic diagnostic manuals, including the DSM, ICD, and MINI, was 9.97% (48 913/900757, 95% CI 8.68–11.35%), with significant evidence of between-study heterogeneity (*Q* = 36 649.12, *τ*au^2^ = 0.0133, *I*^2^ = 99.8%, *p* < 0.001). The summary prevalence of the studies (40 data sets) using the other measures to define subthreshold depression (e.g. self-reported questionnaires) was 13.41% (29 346/233 964, 95% CI 11.45–15.49%), with significant evidence of between-study heterogeneity (*Q* = 7308.44, *τ*au^2^ = 0.009, *I*^2^ = 99.5%, *p* < 0.001). Figure 3 shows a detailed summary of the prevalence of the diagnostic tool.

### Prevalence of subthreshold depression by study-level characteristics

Statistically significant differences in prevalence estimates were found among different age groups (*Q* = 14.32, *p* = 0.008), the youth group having the highest prevalence [6287/32 037, (14.17%, 95% CI 8.82–20.55%)], followed by the elderly group [13 725/10 522, (12.95%, 95% CI 11.41–14.58%)], and the adult group [58 247/997 462, (8.92%, 95% CI 7.51–10.45%)] (see Fig. 2) in studies performed in the United States [30 757/274 144, (15.36%; 95% CI 12.66–18.26%)] compared with those performed outside the United States [47 502/860 577, (8.99%, 95% CI 7.77–10.28%)] (*Q* = 14.51, *p* = 0.001). When the studies were further stratified by continent or region, there were statistically significant differences among different regions (*Q* = 274.16, *p* < 0.001), with North America showing the highest prevalence (15.36%, 95% CI 12.66–18.26%) (see Fig. 2 and online Supplementary Fig. S2 in the supplemental data).

Meta-analytic analysis between cross-sectional studies [57 725/686 501, (11.01%, 95% CI 9.6%–12.5%)] and longitudinal studies [20 534/448 220, (11.1%, 95% CI 8.46–14.05%)] did not reveal statistically significant differences (*Q* = 0.00, *p* = 0.951). Moreover, prevalence estimates from the primary-care samples [759/4752, (12.50%, 95% CI 6.85–19.54%)] and that from the community-based samples were similar [76 741/1 125 217, (10.89%, 95%CI 9.61–12.22%)] (test for subgroup differences, *Q* = 0.35, *p* = 0.618) (see Fig. 2).

When evaluated by Newcastle-Ottawa criteria, no significant difference in prevalence estimates between low-risk (> 3) [64 545/806 081, (11.43%, 95% CI 9.97–12.98%)] and high-risk studies (⩽ 3) [13 714/328 640, (9.74%, 95%CI 7.33–12.45%)] were found. However, after a thorough item analysis, significant differences were observed between studies with representative participant populations [77 010/1126459, 10.5% (95% CI 9.28–11.28%)] and those with less representative participant populations [1249/8262, 15.97% (95% CI 12.25–20.01%)] (*Q* = 7.46, *p* = 0.006; Fig. 4). Beyond the sample representativeness, significant differences were also found in sample size (*Q* = 8.93, *p* = 0.002) and in the validity of the ascertainment of depression (*Q* = 10.30, *p* = 0.001). There were no statistically significant differences in prevalence estimates when studies were stratified by the respondent and nonrespondent comparability or by the thoroughness of the descriptive statistics used for reporting (*p*_s_ > 0.05).

### Incidence risk ratio of individuals with subthreshold depression developing into major depression

Among the 25 longitudinal studies (see online Supplementary Table S1 in the supplemental data), six studies (Broadhead, Blazer, George, & Tse, 1990; Hill, Pettit, Lewinsohn, Seeley, & Klein, 2014; Horwath, Johnson, Klerman, & Weissman, 1992; Klein, Shankman, Lewinsohn, & Seeley, 2009; Laborde-Lahoz et al., 2015; Oh et al., 2021) were excluded when estimating of IRR, as they contained insufficient data for IRR estimation. Therefore, 88 882 individuals from 19 studies were entered into the final analysis (see online Supplementary Table S2 in the supplemental data). Among these studies, most commenced follow-up in the 1980s or early 1990s, and the follow-up period length ranged from 1.0 to 17.5 years, with a mean of 5.29 years and a standard deviation of 4.89.

**Fig. 5.**
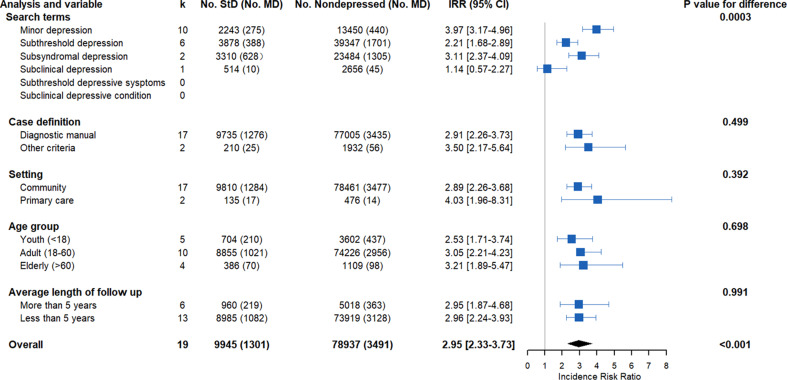
Meta-analysis of the IRR of subthreshold depression developing into major depression under different subgroup analyses. StD, subthreshold depression; MD, major depression; IRR, incidence risk ratio.

When the 19 studies that reported the risk of individuals with subthreshold depression developing major depression were pooled, the results indicated that the transition probability of subthreshold depression to major depression (17.62%) was significantly larger than among non-depressed people (6.08%) (*t* = 3.52, *p* = 0.001, see online Supplementary Fig. S3 in the supplemental data). Further analysis of the incidence rate of the 19 studies indicated that individuals with subthreshold depression had an increased risk of developing major depression (IRR = 2.95, 95% CI 2.33–3.73, *Z* = 9.05, *p* < 0.001, *τ*au^2^ = 0.009, *I*^2^ = 86.2%) (see Fig. 5, online Supplementary Fig. S4, and Tables S6 and S7 in the supplemental data). Different terms mapping subthreshold depression showed a significant difference in IRR (*Q* = 18.60, *p* < 0.001, Fig. 5), the term minor depression had the highest IRR (3.97, 95% CI 3.17–4.96, tau^2^ = 0.027, *I*^2^ = 24.1%), followed by subsyndromal depression (3.11, 95% CI 2.36–4.09, tau^2^ = 0.025, *I*^2^ = 56.1%). Further subgroup analyses estimated similar IRRs for the youth, adult, and elderly and groups, as well as for community-based and primary care samples. Sensitivity analyses demonstrated that baseline results were robust to different sources of study heterogeneity (see online Supplementary Table S8 in the supplemental data). Moreover, pooled IRR estimates across the studies with different follow-up periods (> 5 years *v.* < 5 years, *Q* = 0.01, *p* = 0.99) were not significantly different, suggesting that individuals with subthreshold depression are at a stable risk for developing major depression.

### Prevalence of major depression

The reporting prevalence of major depression in the 117 data sets ranged from 0.71% to 30.99%. Pooling the prevalence estimates for major depression from all included studies yielded a summary prevalence of 6.95% (94 840/1 058 542 individuals; 95% CI 6.29–7.63%), with significant evidence of between-study heterogeneity (*Q* = 19 463.52, *τ*au^2^ = 0.005, *I*^2^ = 99.4%, *p* < 0.001). Further stratified meta-analysis of the age groups, sample sources, and continent or region is shown in online Supplementary Table S9 and Figs S5–S7 in the supplemental data.

### Analysis of publication bias

We performed a two-step publication bias check. First, our visual inspection of the funnel plots of studies reporting on subthreshold depression (online Supplementary Fig. S8 in the supplemental data) and major depression (online Supplementary Fig. S9 in the supplemental data) revealed significant asymmetry, whereas the funnel plots of studies reporting the risk of subthreshold depression developing into major depression (online Supplementary Fig. S10 in the supplemental data) showed minimal asymmetry. Second, we performed Egger's test to identify evidence of publication bias, with smaller studies yielding more extreme estimates for the prevalence of subthreshold depression and major depression (subthreshold depression, *t* = 5.03, *p* < 0.001, major depression, *t* = 2.79, *p* = 0.006). No significant publication bias was identified in estimating the risk of individuals with subthreshold depression developing major depression (*t* = 0.41, *p* = 0.685).

## Discussion

To the best of our knowledge, this is the first study to conduct a comprehensive quantitative review of the estimates of the prevalence of subthreshold depression and the IRR of subthreshold depression developing into major depression simultaneously across the lifespan, covering 1 129 969 individuals in 39 countries. Our results showed that 11.02% (range, 1.50–41.27%) of individuals screened positive for subthreshold depression and that the youth group had the highest prevalence (14.17%), followed by the elderly group (12.95%,) and the adult group (8.92%). To be noticed that, even several terms were used to characterize subthreshold depression, no significance difference in estimating the prevalence among the terms was found. A further meta-analysis of the 19 longitudinal studies reporting the developed outcome of subthreshold depression showed that individuals with subthreshold depression were approximately three times more likely than non-depressed people to develop major depression without difference among the age groups. Terms using minor depression gained the highest IRR compared with other terms. In addition, further analysis revealed that the prevalence of major depression was 6.95%. These findings offer evidence that depression is better expressed as a spectrum on which subthreshold depression coexists with major depression. Furthermore, effective preventive interventions for individuals with subthreshold depression could reduce their risk of developing major depression.

### Different terms tapping similar outcome of subthreshold depression

Our results showed no significant difference among the terms in the prevalence of subthreshold depression, however the term minor depression had the highest IRR. On the one hand, the similar prevalence in tapping subthreshold depression suggests that depression might be a continuous entity. Studies have demonstrated that the impact on health status does not differ significantly between levels of depression (Ayuso-Mateos, Nuevo, Verdes, Naidoo, & Chatterji, 2010; Jeuring, Huisman, Comijs, Stek, & Beekman, 2016) Moreover, there is a trend in diagnostic systems which suggests that psychopathology could be better characterized as dimensions [e.g. Hierarchical Taxonomy of Psychopathology (HiTOP)] (Kotov et al., 2017, 2021) and that depression is a latent continuous variable rather than a categorical entity. In such case, a dimensional perspective tracking the full-blown episodes of depression is encouraged. On the other hand, the IRR results might reflect the critical importance of the tools used for screening subthreshold depression. Minor depression was defined according to diagnosis criteria (i.e. DSM criteria), while for subthreshold depression, also called subclinical depression or subsyndromal symptomatic depression, between 2 and 5 depressive symptoms were required for the diagnosis, and a minimum duration of 2 weeks. Herein, it is important to use the optimal tool for assessing depression among the general population. A large majority of individuals with subthreshold depression are likely to first seek help in primary care and they are likely to form the bulk of persons with depression seeking care (Wang et al., 2007). Sensitizing primary care providers to these conditions would both help in early recognition of depression using an optimal tool, delivery of interventions, both pharmacological and interpersonal or problem-solving therapies, and perhaps in the identification of persons who are at the highest risk of worse outcomes in the future.

### Age-associated difference in estimating the prevalence of subthreshold depression

The present analysis builds on works that demonstrated that subthreshold depression is a risk factor for developing major depression among adults (Tuithof et al., 2018), young people (Wesselhoeft, Sørensen, Heiervang, & Bilenberg, 2013), and the elderly (Cohen, Goh, & Yaffee, 2009). In the current study, we found that subthreshold depression and major depression were prevalent in all age groups, and they were more common in adolescents and less so in adults. Studies have shown that the interactions among familial and genetic, developmental factors, sex hormones, and psychosocial adversity increase the risk of youths experiencing depression (Thapar, Collishaw, Pine, & Thapar, 2012). Compared with adults, negative family relationships, peer victimization through bullying, and maltreatment have more profound influence on youths (Twenge, Cooper, Joiner, Duffy, & Binau, 2019). Moreover, with the proliferation of technology, digital media have transformed childhood, which brings some serious threats like cyberbullying. Thus, it is not surprising that youths had the highest rate of experiencing depression compared with the other two groups.

However, depression in youths has been linked with suicidality (Shaffer & Pfeffer, 2001), functional impairment (Balázs et al., 2013; Steger & Kashdan, 2009), and several negative health outcomes in adulthood (Alaie et al., 2019; Philipson et al., 2020). Timely preventive intervention to reduce subthreshold depression symptoms may significantly reduce depression's impact on children, adolescents, and their families, and it might prevent the onset of future depressive disorders and other adverse outcomes (Méndez, Sánchez-Hernández, Garber, Espada, & Orgilés, 2021; Thapar, Collishaw, Potter, & Thapar, 2010). Notably, by examining the effects of psychological interventions for subthreshold depression in 12 studies, Cuijpers et al. (2021a) revealed that interventions for subthreshold depression are effective for managing the symptoms and are possible indications for preventing the onset of major depression at follow-up in adolescents. Importantly, with the development of psychiatric services for young people, youths experiencing psychological distress get more attention from multiple sources, including family, school, and government, which will further block the progress of subthreshold depression developing into major depression. These might explain why there was no significant difference among age groups in terms of the IRR characterizing the risk of subthreshold depression developing into major depression. The current results indicate that timely interventions might reduce the risk of subthreshold depression developing into major depression.

### Gender difference in estimating the prevalence of subthreshold depression

The summary estimates of subthreshold depression in females (13.8%) are significantly higher than in males (9.68%). Thus, females have a higher risk of experiencing subthreshold depression compared with males. This figure is similar to the findings on major depression. Salk, Hyde, and Abramson (2017) performed a meta-analysis of data from 95 articles covering 1 922 064 individuals and found that females have approximately twice the number of major depressive episodes compared with males. In addition, previous studies (Bennett, Ambrosini, Kudes, Metz, & Rabinovich, 2005; Crockett et al., 2020; González & Vives, 2019) demonstrated gender differences in symptom frequencies in depressed adults, with females having more depressed mood, appetite and sleeping problems, fatigue, anhedonia, and diurnal variation than males did. However, males reported more alternative symptoms than females, such as alcohol and drug abuse, risk-taking, and poor impulse control, which suggests that it is necessary to determine what constitutes depression in women and men. Therefore, gender differences in depression might represent a major health disparity. The long-term outcome magnitude difference of subthreshold depression between females and males needs to be addressed in future research.

### Insights of future research

The findings of this study offer clinical and research insights for the future. First, the current findings provide evidence that depression should be treated as a spectrum rather than as separate categories focusing on the absence or the presence of a full-blown disorder (Benvenuti et al., 2015; Bowins, 2015; McElroy et al., 2021). Studies have shown that depressed mood and irritability are among the most frequent symptoms of subthreshold depression and major depression across studies (Fava et al., 2010; Wesselhoeft, Heiervang, Kragh-Sørensen, Juul Sørensen, & Bilenberg, 2016) and that depressed mood may be optimal for predicting future major depression arising from subthreshold depression (Cuijpers, Beekman, Smit, & Deeg, 2006; Van Voorhees et al., 2008). To identify intervention targets of and to measure the direct clinical implications, efforts to identify sensitive tool to screen subthreshold depression among the general population, quantify risk factors and unravel the exact pathways through which these risk factors affect the course of subthreshold depression are urgently required.

Second, subthreshold symptoms represent promising targets for preventive and early interventions for depression. Subgroup analyses showed similar IRR for different age groups (youth, adult, and elderly), sample type (community and primary care), and years of follow-up (> 5 years and < 5 years), suggesting that the development of subthreshold depression into major depression is a stable feature. Therefore, future work targeting subthreshold depression symptoms might reduce the risk that subthreshold depression will develop into major depression. Studies have indicated that the psychological treatment of subthreshold depression can prevent the onset of a depressive disorder (Hetrick, Cox, Witt, Bir, & Merry, 2016; Myers, Rockhill, & Cortese, 2021). Depending on one's perspective, this might be considered early treatment or indicated prevention, a proactive type of intervention shown to be clinically effective and cost-effective. Those at the highest risk of progression from subthreshold depression to a full-blown depressive disorder would benefit most from treatment. Therefore, timely identification of risk factors associated with such a progression is desirable. For example, studies have examined the effect of preventive intervention programs and brief psychotherapy sessions for adolescents with subthreshold depression and have found evidence that it is possible to reduce the number of new cases of major depression (Cuijpers et al., 2021b; van Zoonen et al., 2014). Similarly, counseling programs in schools and primary care facilities could offer a way of addressing clinically relevant subthreshold depression (Van Voorhees, Melkonian, Marko, Humensky, & Fogel, 2010) while reducing the risk of subsequent major depression among adolescents. Third, based on the results of this study, we recommend considering a gender-informed approach to preventive and early interventions for depression, given that some clinical features and associated factors differ between females and males. For example, Crockett et al. (2020) found that girls displayed a higher rate of depressive mood and sleep problems, whereas boys had greater anhedonia, problems related to concentration, and psychomotor dysregulation. Therefore, when designing prevention intervention programs, gender differences should be taken into account.

### Strengths and limitations

In addition, during the data extraction, the two authors (RZ and XP) extracted the vital characteristics of each paper independently by using a predefined format in Epidata to ascertain the accuracy and reliability of the extracted data. During further checking of the extracted data by the two authors, if any discrepancies were found, the extracted characteristics of the original articles were discussed further until the authors reached a consensus.

Several limitations should be acknowledged. First, we did not have sufficient data to examine gender differences in the IRR of subthreshold depression. Further research is needed to address whether there are gender differences in the IRR of individuals with subthreshold depression compared to non-depressed individuals based on more representative samples. Researchers should be encouraged to expand the data with detailed characteristics and to share the data under the Open Science framework. Second, our database search in the current study only included articles written in English, which could exclude articles written in other languages such as Chinese. Increasingly, data come from nationally representative samples involving major, costly undertakings. Such data should result in a significant number of publications, at least one of which should be published in English. If such had been the case, we would have included these studies. Third, the factors (e.g. social support, personality) contributing to subthreshold depression developing into major depression still need to be addressed. Within the limits of the included studies, there were insufficient data to profile the factors predisposing individuals to the progression of subthreshold depression to major depression. Future studies might combine data from behavioral, biological, and genetic perspectives, among others, to fully chart the factors contributing to increasing the risk of subthreshold depression progressing to major depression. Fourth, heterogeneity as indicated by I^2^ was very high (>75%) in our current analyses. Several subgroup analyses (e.g. terms mapping subthreshold depression, age group, etc.) were performed, but none of them could explain such a high level. This indicates that the effects differed considerably across the included studies, and it is not clear what caused these differences, therefore the results of the current study should be taken with some caution. Last but not least, subthreshold depression in the current study was screened either by using diagnostic criteria (e.g. DSM) or continuous depressive symptoms inventories with arbitrary cut-offs. Recently, there is a trend in diagnostic systems which suggest that psychopathology could be better characterized as dimensions (e.g. HiTOP) (Kotov et al., 2017, 2021). This view considers depression as a latent continuous variable rather than a categorical entity. Under such framework, depressive symptoms might fluctuate over time and depressive traits also change but more slowly, so a trait level measurement may be conceptualized as a moving average of the corresponding symptoms (Wright & Simms, 2016). In future study, it will be worth exploring how depressive traits contribute to the IRR of subthreshold depression developing into a major depression by using a longitudinal design.

## Conclusion

This meta-analysis is a thorough synthesis of 113 studies with data involving 1 129 969 individuals from 39 countries. A comprehensive quantitative review of data on the summary estimates of subthreshold depression and the IRR of subthreshold depression developing into major depression across the lifespan were conducted. The prevalence of subthreshold depression among the general population is about 11%. Importantly, individuals with subthreshold depression were approximately three times more likely than non-depressed people to develop major depression across all age groups. These findings offer evidence that depression is better expressed as a spectrum on which subthreshold depression coexists with major depression and that effective preventive interventions for individuals with subthreshold depression could be useful in decreasing the risk of developing major depression. The identification of cost-efficient and long-lasting interventions is encouraged to prevent the progression of early subthreshold depression into major depression.

## Supplementary material

For supplementary material accompanying this paper visit https://doi.org/10.1017/S0033291722000241. click here to view supplementary material
