# Supplementary material for: The prevalence and risk of developing major depression among individuals with subthreshold depression in the general population
Source: Psychol Med. 2022 Feb 14;53(8):3611–20. doi: 10.1017/S0033291722000241 (PMC10277767; doi:10.1017/S0033291722000241)
Supplement: Supplementary file 1 [file S0033291722000241sup001.zip › S0033291722000241sup001.pdf]

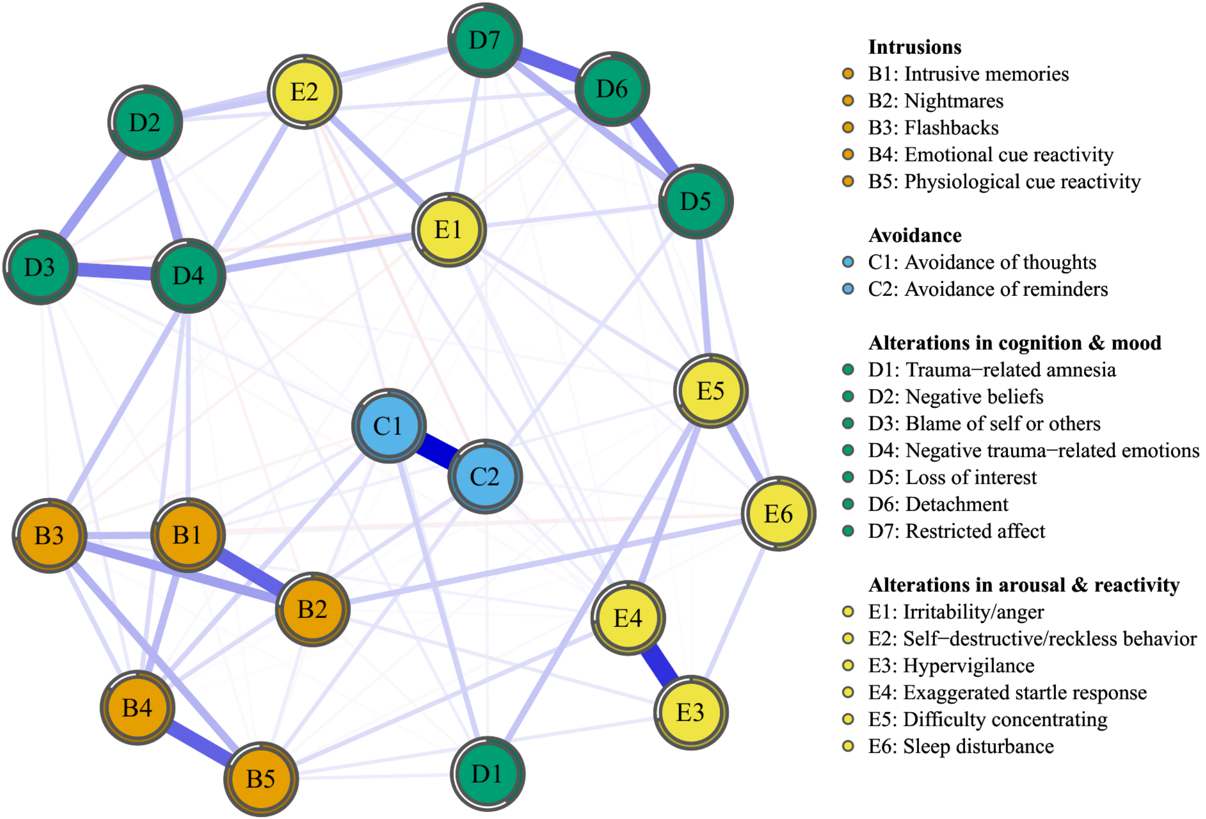

Supplemental Figure S1. Full Between-Subjects Person-mean Nework. All estimated edges are depicted. Circles around nodes reflect symptom predictability and can be interpreted as variance in the symptom that can be accounted for by the rest of the network. Node location has been averaged across the person-mean and slope networks.
