# Supplementary material for: The prevalence and risk of developing major depression among individuals with subthreshold depression in the general population
Source: Psychol Med. 2022 Feb 14;53(8):3611–20. doi: 10.1017/S0033291722000241 (PMC10277767; doi:10.1017/S0033291722000241)
Supplement: Supplementary file 1 [file S0033291722000241sup001.zip › S0033291722000241sup003.pdf]

Supplemental Figure 3. Reduced Between-Subjects Person-mean Network

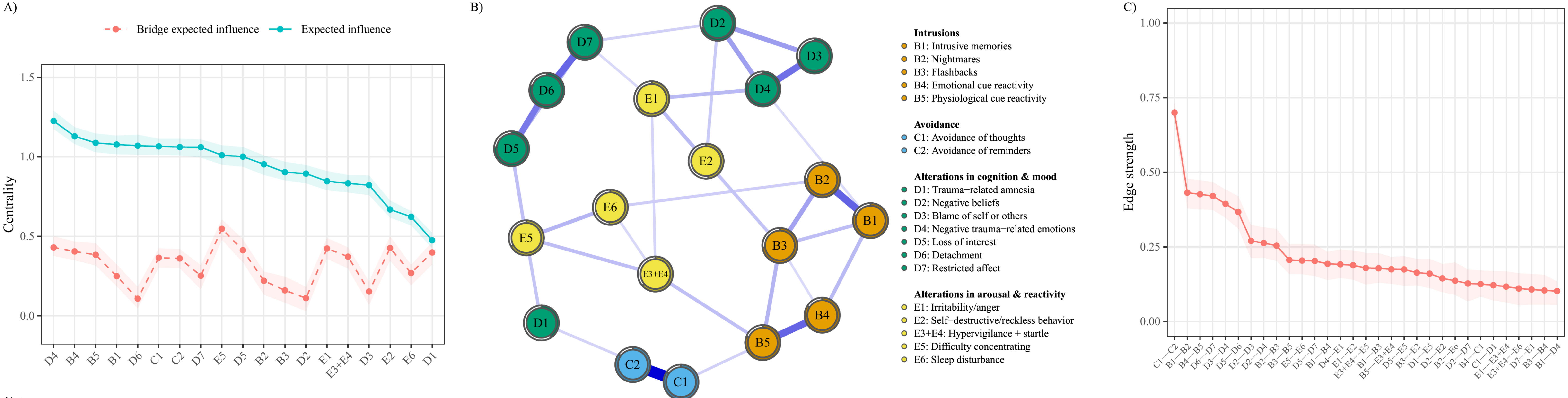

Note.
A) Node expected influence plot. Shaded areas reveal 95% CI. Expected influence values reflect all estimated edges. B) Network model. Figure displays only edges with a magnitude greater than .1. Circles around nodes reflect symptom predictability and can be interpreted as variance in the symptom that can be accounted for by the rest of the network. C) Edge Strength. Shaded area reveals 95% CI. All edges depicted in the network model (panel B) are included.
