# Supplementary material for: The prevalence and risk of developing major depression among individuals with subthreshold depression in the general population
Source: Psychol Med. 2022 Feb 14;53(8):3611–20. doi: 10.1017/S0033291722000241 (PMC10277767; doi:10.1017/S0033291722000241)
Supplement: Supplementary file 1 [file S0033291722000241sup001.zip › S0033291722000241sup004.pdf]

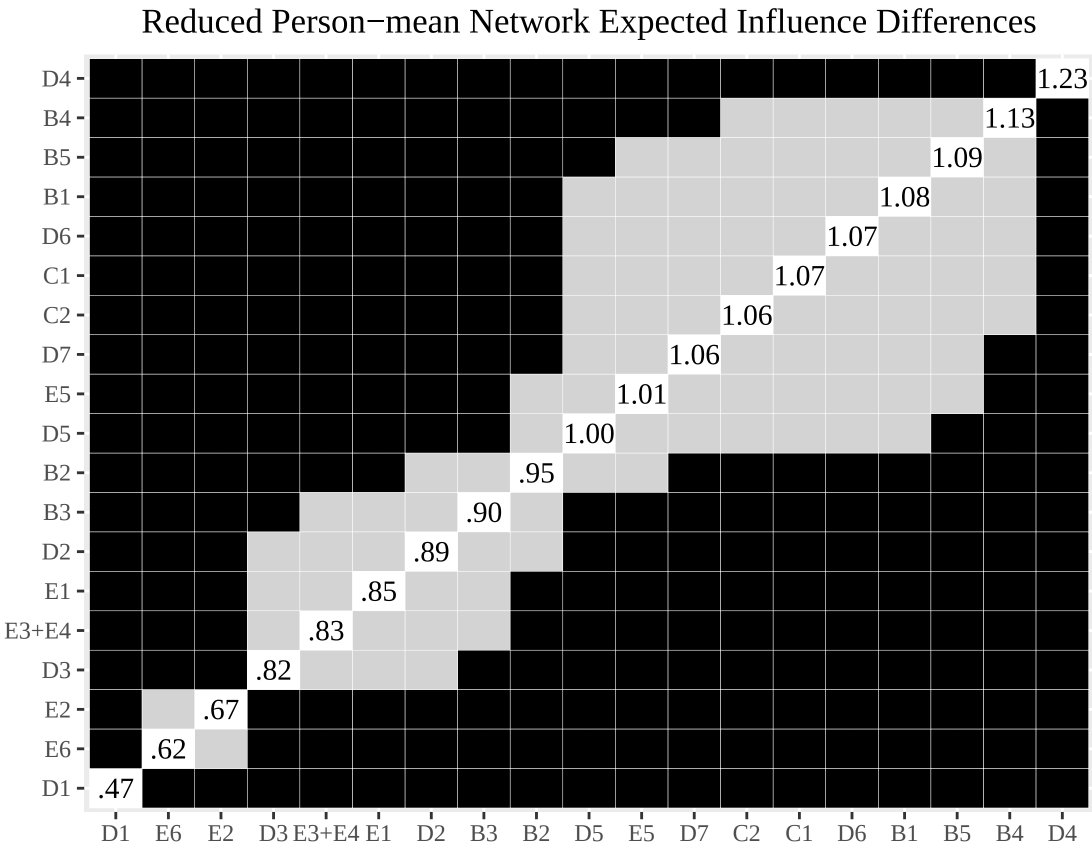

Note. Difference tests based on nonparametric bootstrapping. Black cells indicate significant difference ($p < .05$) between estimates. Plot diagonals display the observed expected influence for each node.
