# Supplementary material for: The prevalence and risk of developing major depression among individuals with subthreshold depression in the general population
Source: Psychol Med. 2022 Feb 14;53(8):3611–20. doi: 10.1017/S0033291722000241 (PMC10277767; doi:10.1017/S0033291722000241)
Supplement: Supplementary file 1 [file S0033291722000241sup001.zip › S0033291722000241sup006.docx]

**Supplemental Appendix. PCL-5 Topological Overlap**

**Method**

An analysis of topological overlap was conducted for both the person-mean (i.e., intercept) network and slope network. Topological overlap was empirically examined using the *goldbricker* function, which is implemented in the *networktools* package (Jones, 2021). Levinson and colleagues (2018) provide an introduction to the goldbricker function and its use. In brief, the goldbricker approach identifies all nodes in the proposed network with zero-order correlations above a provided threshold as possible “bad pairs” (i.e., redundant content). Possible bad pairs are then evaluated by comparing their correlations with the rest of the nodes in the network. The rationale being that if identified “bad pairs” do represent the same underlying construct, they should have correlations of comparable magnitude with the other variables in the network. The proportion of significantly different correlations was used to determine if the amount of topological overlap was sufficient enough to require removal.

Zero-order correlations greater than .7 (i.e., roughly 50% shared variance) were identified as possible “bad pairs” for the current analyses. A threshold of .25 and p-value of .01 was selected for item removal. That is, possible “bad pairs” of items had to have at least 25% of their correlations with third variables in the network be statistically different (at p < .01) in order to be considered non-redundant. In line with the recommendations of Hittner and colleagues (2003), Williams’s (1959) standard *t* formula was used to compare the dependent correlations.

**Results**

In the person-mean network 45 out of 190 item pairs were identified with zero-order correlations greater than .7. Of those, only one pair (items 17 and 18; hypervigilance and hyperstartle) had comparable associations with at least 75% of the other nodes in the network. Only 15% of the correlations were statistically different. The first principal component of the two variables was estimated and used in place of the two overlapping variables. All primary analyses on the person-mean network were replicated using this reduced item set. Results are provided in Supplemental Table S4 and Supplemental Figures S3 and S4.

The goldbricker function indicated there were no redundant item pairs in the between-subject slopes network.

**References**

Hittner, J. B., May, K., & Silver, N. C. (2003). A Monte Carlo evaluation of tests for comparing dependent correlations. *The Journal of General Psychology*, *130*(2), 149–168. https://doi.org/10.1080/00221300309601282

Jones, P. (2021). *Package “networktools”* (1.4.0) [Computer software]. https://CRAN.R-project.org/package=networktools

Levinson, C. A., Brosof, L. C., Vanzhula, I., Christian, C., Jones, P., Rodebaugh, T. L., Langer, J. K., White, E. K., Warren, C., Weeks, J. W., Menatti, A., Lim, M. H., & Fernandez, K. C. (2018). Social anxiety and eating disorder comorbidity and underlying vulnerabilities: Using network analysis to conceptualize comorbidity. *International Journal of Eating Disorders*, *51*(7), 693–709. https://doi.org/10.1002/eat.22890

Williams, E. J. (1959). The Comparison of Regression Variables. *Journal of the Royal Statistical Society. Series B (Methodological)*, *21*(2), 396–399.
